# Hypertensive Disorders in Pregnancy at the John F. Kennedy Maternity Center (JFKMC), Liberia: Burden, Sub-Types, and Maternofetal Outcome

**DOI:** 10.4314/ejhs.v33i4.6

**Published:** 2023-07

**Authors:** Williams O. Odunvbun, Billy C. Johnson, Daniel G. Urey

**Affiliations:** 1 Department of Obstetrics and Gynecology, John F. Kennedy Maternity Center, Monrovia. Liberia; 2 Department of Obstetrics and Gynecology, Faculty of Clinical Medicine, College of Health Sciences, Delta State University, Abraka, Delta State. Nigeria

**Keywords:** Hypertensive disorders, prevalence, maternal fatality rate, perinatal fatality rate, outcome measure

## Abstract

**Background:**

Hypertensive disorders in pregnancy (HDP) are a leading cause of maternal and fetal death, especially in a resource-constrained setting. There is no study from Liberia on the disorder. This pilot study aimed to determine the burden, sub-types, and maternal-fetal outcomes of hypertensive disorders in pregnancy at the John F. Kennedy Maternity Center (JFKMC), Liberia.

**Methods:**

From January 1 to December 31, 2020, the medical records of 130 pregnant and post-partum patients admitted with Hypertensive disorders in pregnancy (HDP) in a census method of sampling were retrieved, while 83.1% (108) were suitable for analysis in an institutional cross-sectional retrospective study in the department of obstetrics and gynecology at the John F. Kennedy Maternity Center, Liberia. The extracted information was analyzed using SPSS version 26. Results were presented in frequencies and percentages. The statistical association between categorical variables was subjected to the Chi-square test. The level of significance was set at a P-value of < 0.05

**Results:**

There was an institutional prevalence of 3.0% of HDP. The maternal fatality rate was 12.3%, while the perinatal fatality rate was 14.3%. There was a significant association between HELLP syndrome and Severe pre-eclampsia with maternal death, P< 0.001. Prematurity, first minutes Apgar score <5, NICU admission, and low birth weight were associated with perinatal deaths (P <0.001)

**Conclusion:**

HDP was an important contributor to maternal and perinatal deaths at the JFKMC, Liberia. Continuous support by the government and development partners for the provision of critical life-saving medical equipment at the JFKMC is recommended.

## Introduction

Hypertensive disorders in pregnancy remain an important and leading contributor to poor maternal and fetal outcomes in pregnancy globally, and in particular, in resource-constrained developing countries (1). Six to twelve percent of all pregnancies are complicated by hypertension (2). The spectrum of disorders described as HDP, according to the National Institute of Health and Care Excellence: included Gestational Hypertension, in which hypertension presents in pregnancy after 20 weeks without proteinuria; Pre-eclampsia: new hypertension presenting after 20 weeks with significant proteinuria; Chronic hypertension: hypertension present at the booking visit or before 20 weeks or if the woman is already taking antihypertensive medication when referred to maternity services, which may be of primary or secondary etiology; Eclampsia: a convulsive condition associated with pre-eclampsia; HELLP syndrome: hemolysis, elevated liver enzymes and low platelet count; Severe pre-eclampsia: pre-eclampsia with severe hypertension and/or with symptoms, and/or biochemical and/or hematological impairment; Hypertension with or without proteinuria but with insufficient information to classify it (3).

Hypertension in pregnancy refers to a systolic blood pressure of ≥140 mmHg or diastolic blood pressure of ≥ 90 mmHg (4). NICE (3) defines significant proteinuria as urinary protein/creatinine ratio greater than 30 mg/mmol or validated 24-hour urine collection result showing greater than 300 mg protein, or proteinuria of >2 + and signs and symptoms of severe pre-eclampsia (5).

The burden of disease is a measure that assesses and compares the relative impact of different diseases or disorders on a population by quantifying health loss due to illness (or conditions) that remains after prevention, treatment, or rehabilitation efforts of the health system and society. For HDP, the burden is not well documented in several countries globally, and a few countries have data on their incidence (6). In a systematic review and meta-analysis (7) that examined the prevalence of hypertensive disorders of pregnancy and pregnancy outcomes in sub-Saharan Africa (SSA), in which observational facility-based studies, irrespective of publication status, sample size, language, and follow-up duration from 19 countries between 2000 and 2018, there was no single cited publication on HDP from Liberia. The Liberia Demographic and Health Survey (LDHS) 2013(8) reported that HDP was the second leading cause of maternal death after hemorrhage in Liberia. To the researchers' best knowledge, there was no published article in the literature on the burden of HDP from Liberia. This facility-based study may therefore be considered a pilot work on HDP from Liberia. It aimed to determine the burden, sub-types, and maternal and fetal outcomes of HDP at the JFKMC during the first wave of the Covid-19 pandemic in West Africa. The main outcome measure was maternal and perinatal fatalities from HDP at the facility.

## Methods and Materials

**Study area and study period**: This study on HDP was conducted at the Maternity Center of the only tertiary health facility in Liberia, the JFKMC. Liberia is one of the countries in the West Africa Sub-region. It had a population of 4,694,608 by the 2008 National population census (9). The world Bank, however, estimated the population in 2021 to be 5,180,208 (10). The country is made up of 15 Counties. Superintendents appointed by the President of Liberia administer the Counties. The counties are home to 16 indigenous ethnic groups: Kpelle, Bassa, Mano, Gio, Kru, Grebo, Krahn, Vai, Gola, Madingo, Mende, Kissi, Gbandi, Americo-Liberian, Dei, and Belleh.

In addition to the JFKMC, there are seven mission hospitals and county health facilities in Liberia. The various health facilities are under the supervision of the Ministry of health. The government of Liberia operates free maternity services. The lack of funds from the central government impacted the county facilities' capacity to meet the populace's health needs.

Consequently, many obstetric cases with complications were referred to the JFKMC in the Capital City of Monrovia, which often resulted in congestion at the facility. This congestion was made worse by the Covid -19 pandemic because many health facilities were depleted of staff, who were either infected or seconded to assist in managing patients at the various Covid-19 Isolation Centers. The services at JFKMC were out-of-pocket payments, making it difficult for the poor sometimes to access its services. The minimum wage for Civil Servants in Liberia was equivalent to $32.94, while unskilled laborers earned $0.09 per hour (11).

The World Health Organization (WHO), the World Bank, and other development partners have continued to assist the Liberian health sector in capacity building, especially in attracting consultants in various specialties from other African countries and beyond. Despite this support, there was still a wide gap in infrastructure needs in the health sector. For example, until the last quarter of 2021, there was no standard Intensive care unit in any of the health facilities in Liberia, no ventilators, and no available dialysis unit. These were essential requirements for managing complications from HDP, and their lack could adversely affect the maternal outcome of the disorder. This study was conducted from January 1, 2020, to December 31,2020.

## Methods

**Design**: This was a cross-sectional retrospective study of patients admitted with the diagnosis of HDP in a census sampling method.

**Study population**: All the patients admitted and managed for HDP at the JFKMC who satisfied the inclusion criteria.

**Inclusion and exclusion criteria**: All pregnant and post-partum patients who presented with diagnostic criteria of HDP. These included obstetric patients with gestational hypertension, pre-eclampsia, chronic hypertension, eclampsia, severe pre-eclampsia, and hypertension ± without proteinuria but with insufficient information to classify it. While all obstetric patients with seizure disorders, prenatally or post-partum, without diagnostic features of pre-eclampsia, were excluded from the study, which included patients with hypoglycemia, diabetic ketoacidosis, and electrolyte derangements.

**Data collection tools and the procedure**: The names and hospital numbers of all the patients with the diagnosis of HDP were copied by the researchers from the admission notebook at the JFKMC triage, labor ward, and labor ward theater. The medical records of all the patients with HDP, including neonatal outcomes, were retrieved by the staff of the record department. The relevant information on the medical records was transferred to the study proforma, which was first subjected to content validity by a review of senior faculty staff, followed by a pilot study using the same study population. The information extracted included booking status, socio-demographic and clinical characteristics, the sub-type of HDP, mode of delivery, maternal mortality, stillbirths, early neonatal death, gestational age at delivery, Apgar score at 5 minutes, NICU admission, and fetal birthweight. The information on the proforma was subsequently transferred to a computer-based data sheet for analysis.

**Data quality control**: All extraction form was examined for the completeness of data entered by the lead researcher before transfer to the computer data set.

**Data processing and Analysis**: The computer data set was examined and cleaned up before analysis by SPSS version 26.0 (IBM Inc, Chicago, IL, USA). Dataset was presented in frequencies and percentages. The statistical association between categorical variables was subjected to Chi-square. The level of significance was set at a p-value of <0.05.

**Ethical approval**: Approval was obtained for the study from the Institutional Research Board (IRB), JFKMC. Approval ID: #2022/05/JFK0097. The content and identity of all medical records used for the study were confidential. They were used only for the study.

## Results

The medical records of 130 patients satisfied the inclusion criteria, of which 83.1% (108/108) were available for analysis. Seven case notes could not be retrieved, while poor documentation in the remaining fifteen case notes made them unsuitable for analysis.

During the study period, there were 4328 live births at the maternity, with cesarean section accounting for 43.0% (1,862) of the deliveries. The prevalence of HDP was 3.0%. HDP was the facility's leading cause of maternal death, accounting for 32.0 % (16) of the 50 maternal deaths. The maternal fatality rate was 12.3% from HDP. The perinatal fatality rate was 14.3%.

The mean age ± SD of subjects was 25.2 ± 7.2 years. Their age ranged from 14.0 to 42.0 years. Over a quarter (28.7%) of the subjects were teenagers, with over 50% single. The unmarried adolescents were less likely to avail themselves of antenatal services for financial reasons, with resultant delays in the early recognition of disease complications. Over 3/4 (77.5%) of the subjects had only primary education. Most subjects were either students or unemployed, which may have affected the health-seeking behavior of the subjects financially and delayed presentation at the facility. Christianity was the main (94%) religion among subjects. Less than half (41.7%) of the subjects were booked for antenatal services (Table 1).

**Table 1:** Socio-demographic and reproductive characteristics of Patients with HDP (n=108)

Variable	Frequency	%
Age group		
<20	31	28.7
20 - 24	23	21.3
25 - 29	27	25.0
30 - 34	13	12.0
35 - 39	9	8.3
40 and above	5	4.6
Marital Status		
Married	17	15.7
Single	55	50.9
Divorced	2	1.9
Cohabitating	34	31.5
Educational Qualification		
No formal education	13	12.1
Primary	70	65.4
Secondary	18	16.8
Tertiary	6	5.6
Occupation		
Unemployed	32	29.6
Student	44	40.7
Civil service	1	0.9
Petty trading	30	27.8
Professional	1	0.9
Religion		
Christianity	94	87.0
Moslem	14	13.0
Atheist	0	0.0
Others	0	0.0
Booking Status		
yes	45	41.7
No	56	51.8
Not documented	7	6.5

The study facility's four leading sub-types of HDP were gestational hypertension, pre-eclampsia, eclampsia, and chronic hypertension, with 37.0%, 18.5%, 14.8%, and 14.8%, respectively (Table 2).

**Table 2:** Distribution of sub-types of hypertensive disorders in pregnancy among subjects

Variable	Frequency	Percentage	N=108
Gestational Hypertension	40	37.0	
Pre-eclampsia	20	18.5	
Eclampsia	14	14.8	
Chronic hypertension	16	13.0	
HELLP syndrome	2	1.9	
Severe pre-eclampsia	9	8.3	
Unclassified	7	6.5	

The fatality rate from HELLP syndrome was 100%, followed by 66.7% from severe pre-eclampsia; the two patients with HELLP syndrome developed coagulopathy at presentation. Both were unbooked and died after receiving 8 and 7 units of blood from hemorrhagic shock and cardiac arrest within 24 hours of admission. Seven deaths from severe pre-eclampsia and two from eclampsia were due to pulmonary edema. The duration of admission ranged from 2 to 5 days. The three deaths from cerebrovascular accidents occurred within one week of admission, while the remaining two died from renal complications; the absence of dialysis services contributed to the deaths (Table 3).

**Table 3:** The relationship between sub-types of HDP and maternal mortality

Variable	Maternal mortality			

Sub-type of HDP	No	Yes	χ^2^	p
Gestational hypertension	38(95.0)	2(5.0)	**37.406**	<0.001
Pre-eclampsia	20(100.0)	0(0.0)		
Chronic hypertension	13(81.3)	3(18.8)		
Eclampsia	12(85.7)	2(14.3)		
HELLP Syndrome	0(0.0)	2(100.0)		
Severe Pre-Eclampsia	3(33.3)	6(66.7)		
Hypertension with inadequate	6(85.7)	1(14.3)		
information for classification				

There were more mortalities among the women who did not register for antenatal care compared to those who received antenatal care, 25.0% versus 4.5% (P= 0.005). Antenatal care remains an important health screening platform for early detection, treatment, or counseling on medical and social conditions that may militate against safe motherhood and the delivery of healthy offspring. Failure to access antenatal services would have contributed to complications and late presentation among the unbooked subjects (Table 4).

**Table 4:** The Relationship between booking status and maternal mortality

Variable	Maternal Mortality			
	Yes	No	χ^2^	p-value
Booking status				
Yes	43 (95.5)	2 (4.5)	7.908	0.005
No	42 (75.0)	14 (25.0)		

Over half (52.8%) of the neonates delivered by the subjects were preterm, while over a quarter 25.9%) of them had 5 minutes Apgar score that was less than 7, and they were all admitted into the Neonatal Intensive Unit (NICU). Half (50%) of the neonates had low birth weight, with a mean birth weight ± SD of 2.5 ± 0.7kg. There were 20 perinatal mortalities. Ten twin deliveries were recorded among the subject, which resulted in a perinatal fatality rate of 14.3% (20/140), made up of 4 stillbirths (2.9%) and 16 (11.4%) early neonatal deaths (Table 5).

**Table 5:** The distribution of the clinical characteristics and fetal outcomes of hypertensive disorders in pregnancy

Variable	Frequency	Percentage
Gestational age (weeks)		
<37	57	52.8
≥37	51	47.2
5 mins APGAR score		
<7	28	25.9
≥7	80	74.1
NICU Admission	28	25.9
Birth weight (kg)		
≥1 - <2.5	54	50.0
≥2.5	54	50.0
Mean ± SD (2.5 ±0.7kg)		
Perinatal deaths	20	14.. 3
Stillbirths	4	2.9
Early neonatal deaths	16	11.4

Table 6 showed that preterm deliveries, 5^th^ minutes Apgar score < 7, admission into NICU, and low birthweight were all associated with early neonatal death (P< 0.001). The pediatrics department had five faculty staff, comprising two Liberians and three from the West African Sub-region. The foreign faculties were employed under the funding support of the World Bank and other development partners. The constraints in the neonatal intensive unit included limited space with overcrowding, inadequate incubators, limited oxygen supply, shortage of consumables, and inadequate laboratory support. The payment for neonatal services, like other services in the hospital, was out-of-pocket. Many patients were usually unable to meet with prompt payment for the services, often resulting in delays in intervention, consequently affecting the quality of neonatal services. The contending financial needs of the different sectors of the Liberian economy after decades of war, the Ebola health crisis, and the Covid-19 Pandemic resulted in the lean application of available resources and inadequate budgetary allocation to the health facility. All of the above factors appeared to have negatively impacted the quality of neonatal services at the JFKMC, Liberia. Two of the four stillbirths were delivered to mothers with HELLP syndrome who also died, while the other two were to mothers with severe pre-eclampsia. All sixteen neonates with ENND were admitted into NICU. They all died within a week of admission from complications associated with prematurity and sepsis.

**Table 6:** The relationship between clinical characteristics at birth with perinatal mortality

Variable	Early Neonatal death			
	Yes	No	χ^2^	^p^
Gestational age at birth				
< 37 weeks	16(28.6)	40(71.4)	17.441	<0.001
>37 weeks	0(0.0)	52(100.0)		
5 min Apgar Score				
Less than seven	12(42.9)	16(57.1)	23.554	<0.001
Seven and above	4(5.0)	76(95.0)		
NICU Admission				
Yes	16(57.1)	12(42.9)	53.665	<0.001
No	0(0.0)	80(100.0)		
Birth weight				
<2.5kg	16(30.2)	37(69.8)	19.491	<0.001
≥2.5 kg	0(0.0)	55(100.0)		

## Discussion

This institutional cross-sectional retrospective study was the first on the subject of HDP from Liberia. It may therefore be considered a pilot study. The study aimed at determining the burden, sub-types, and maternofetal outcomes of HDP, at the JFKMC during the first wave of the Covid-19 pandemic in West Africa. The main outcome measure was maternal and perinatal fatality rates from HDP. There was an institutional prevalence rate of 3.0% from HDP. The maternal fatality rate was 12.3%. HDP accounted for 32.0% out of the 50 maternal deaths during the study period. The perinatal fatality rate from HDP was 14.3%.

The prevalence rate in our study was higher than the 1.0%, 2.17%, and 2.6% found in Nigeria, Sudan, and Ethiopia, respectively (12,13,14). Two studies from Ghana (15, 16), a cross-sectional study, and a prospective cohort study reported prevalence rates of HDP that was much higher than the rate in this study, with 19.8% and 11.3%, respectively. .Ebeigbe and Aziken (17) reported a prevalence rate of 6.3% in early onset hypertensive disorder/pre-eclampsia in the neighboring West–African country of Nigeria. The overall prevalence of HDP was 8% in SSA, which is in line with the global estimates of 5.2%-8.2% (18). In a meta-analysis (7), though there was high heterogeneity between studies, it revealed an insignificant decrease in a sub-group analysis. This meta-analysis did not identify any other reason for the heterogeneity. This finding suggested that either the populations with HDP are varied or the diagnosis of HDP and outcomes may not be consistent. For example, the dipstick method (colorimetric reagent strip) was used at the study facility to quantify proteinuria. It is graded from trace to 4+, reflecting the severity of proteinuria and pre-eclampsia. The result from this method may be influenced by urine concentration and inter- and intra-observer errors (19). Other suggested reasons for varied prevalence rates from studies included differences in geographical locations, the sensitivity of disorders' diagnostic and measurement methods, socio-economic status, and sample size. The need to standardize the diagnosis of HDP in sub-Saharan Africa has been recommended (20).

The maternal case fatality rate of 12.3% reported in our study more than doubled the 5.1% reported in a prospective cohort study in Uganda (21) and 5.4% in Ethiopia by Zenebe W. et al. (22). Epidemiological data sourced from WHO suggested that the overall mortality associated with HDP among countries in Africa, Latin America, and the Caribbean did not differ, even though overall mortality was much higher in Africa (23). Some of the factors that contributed to the high mortality rate in this study included socio-demographic variables: The vicious circle of ignorance, poverty, and disease was noted among subjects that died. Education has an important role in the health-seeking behavior of any population. While the primary level of education was considered free in Liberia, the same could not be said of public secondary education, for which the cost of school registration was often unaffordable to a lot of Liberians, especially those residents outside the country's capital of Monrovia, where most of the educational facilities were either privately owned or run by various Christian Missions. The low level of education, high unemployment, and poverty contributed to the fatalities. The decades of war took their toll on several public infrastructures, such as roads and health. The roads leading to the JFKMC, in Monrovia, from many of the Counties were in very poor condition. Patients spent several hours before accessing health services, resulting in delays and complications. The congestion occasioned by the Covid -19 pandemic only aggravated an already difficult situation. The higher mortality observed in developing countries in HDP also resulted from the quality of obstetric care, frequently complicated by infrastructural deficits, with resultant delays in care.

The perinatal fatality ratio of 14.3% in this study was comparable to the 15.0% recorded in a survey conducted in the Ethiopian Tigray region (20) but higher than studies conducted in Ghana, Nigeria, and Madagascar, with 10.6%, 7.6%, 8.7%, respectively (15, 25, 26). The different perinatal outcomes may have been influenced by the quality of NICU services, intrapartum care, and the number of delays before delivery, which could have impacted the severity of neonatal birth asphyxia. Because mothers were made to pay for services, failure or delays in making such payments may have contributed to some of the fatalities. The contribution of the Covid – 19 pandemics also needed to be mentioned. At the pandemic's peak, there was an acute scarcity of oxygen in many countries, including Liberia, leading to the rationing of the life-saving item.

The stillbirth rate of 2.9% was lower than the 6.8% and 5.4% recorded in Ghana and Zimbabwe studies (15, 27). On the other hand, this study documented a 25.9% incidence of birth asphyxia which was higher than the 15.2%, 10.1%, and 21.8% reported in Ghana, Ethiopia, and Uganda, respectively (15, 28, 29). In contrast, reported data from India had an incidence of birth asphyxia of 27.1% (30). The quality of prenatal, intrapartum, and NICU care and the inherent contribution of placenta insufficiency associated with severe HDP, and prematurity are all factors responsible for the different degrees of birth asphyxia.

The strength of this study comes from the facility setting, which was the main recipient of the referrals from the 15 counties during the first wave of Covid -19 pandemic. Thus, findings from this study highlighting the burden of hypertensive disorder at the JFKMC could be explored in future studies on HDP in Liberia. The limitations included the retrospective design, small sample size, and the challenge in case note retrieval at the study facility.

In summary, HDP was an important contributor to maternal and perinatal deaths at the JFKMC, Liberia. The study indicated the need for improved equipment by the government and development partners for the only tertiary health facility in Liberia. Based on the limitations and results, the authors would want to explore a multi-center study to examine the burden of HDP in Liberia in future research, which may assist in advising the government in policy formulation and resource deployment in mitigating the burden of HDP in Liberia.
